# Neonatal humeral fracture and scalp laceration following difficult cesarean delivery for transverse lie: a case report

**DOI:** 10.1515/crpm-2026-0018

**Published:** 2026-09-24

**Authors:** Abdullah Aljasar, Zainab Aljasar, Nedaa Khaiyon

**Affiliations:** Royal College of Surgeons in Ireland (RCSI), Adliya, Bahrain; Department of Obstetrics and Gynecology, Aisha Bint Hamad Al-Alattiyah Hospital, Hamad Medical Corporation, Doha, Qatar

**Keywords:** transverse lie, neonatal birth trauma, humeral fracture, scalp laceration, cesarean section, external cephalic version

## Abstract

**Objectives:**

Transverse lie in advanced labor presents significant obstetric challenges and may complicate fetal extraction during cesarean delivery. Although cesarean delivery is generally considered protective against birth trauma, neonatal injuries may still occur during technically difficult extractions.

**Case presentation:**

A 41-year-old gravida 6 para 5 woman with gestational diabetes mellitus, polyhydramnios, and an unstable transverse lie underwent external cephalic version at 37+3 weeks’ gestation. Although cephalic presentation was initially achieved, the fetus reverted to a transverse lie within 3 h. With cervical dilatation of 8 cm and no presenting part, emergency cesarean delivery was performed under general anaesthesia. A poorly formed lower uterine segment intraoperatively necessitated the use of an inverted T uterine incision, and the neonate sustained a 2 cm scalp laceration and a minimally displaced right humeral shaft fracture. The scalp laceration was repaired immediately, while the humeral fracture was diagnosed on postnatal day 3 following the development of upper limb weakness and an absent Moro reflex. Both injuries were managed successfully with favourable outcomes.

**Conclusions:**

This case highlights the challenges of cesarean delivery for transverse lie in advanced labor and demonstrates that neonatal trauma may occur despite operative delivery. Careful surgical planning, experienced obstetric involvement, and thorough neonatal assessment following difficult extraction are essential.

## Introduction

Neonatal birth trauma is traditionally associated with difficult vaginal delivery; however, injury can also occur during cesarean delivery, particularly when fetal extraction is technically challenging [1], 2]. Although cesarean section generally reduces exposure to the compressive, rotational, and tractional forces encountered during vaginal birth, difficult extraction may still require substantial fetal manipulation, increasing the risk of injury [1], [2], [3].

Several obstetric factors increase the likelihood of difficult fetal extraction during cesarean delivery. These include advanced labor, malpresentation, impacted fetal parts, inadequate access through a poorly developed lower uterine segment, and the need for non-standard uterine incisions [4]. In situations like this, performing an extension of the uterine incision and increased traction may be necessary to achieve delivery, which could potentially expose the fetus to significant mechanical forces [4], 5].

Transverse lie is an uncommon malpresentation associated with substantial challenges during labor and delivery. When accompanied by advanced cervical dilatation, extraction can be particularly difficult because the fetus is not aligned with the uterine incision and access to the presenting part may be limited [6]. Consequently, operative delivery may require complex manoeuvres and additional uterine incisions to facilitate delivery.

We report a case of neonatal humeral fracture and scalp laceration following emergency cesarean delivery for transverse lie in advanced labor. This case highlights the mechanisms by which neonatal injury may occur during difficult fetal extraction and underscores the importance of careful postnatal assessment following technically demanding operative deliveries.

## Case presentation

A 41-year-old woman, gravida 6 para 5, with 5 previous uncomplicated vaginal deliveries, was followed in a hospital setting with 4 documented antenatal visits during her current pregnancy. At 34 weeks of gestation, she was diagnosed with gestational diabetes mellitus. Additionally, she was also diagnosed with iron deficiency anemia with a reported haemoglobin level of 6.8 g/dL, where the patient denied blood transfusion and instead was managed with intravenous iron therapy.

At 35+2 weeks, her blood glucose levels remained uncontrolled, and metformin therapy was initiated. Ultrasound at that time demonstrated an amniotic fluid index of 19 cm with a transverse lie, and by 36+6 weeks, her blood glucose levels were satisfactorily controlled.

At 37+3 weeks, ultrasound showed breech presentation with an amniotic fluid index of 22.2 cm and a maximum vertical pocket of 8.9 cm. The patient was admitted for a possible induction of labor in case of a cephalic presentation. However, a repeated bedside ultrasonography was done after admission, which confirmed a transverse lie. Following counselling regarding external cephalic version vs. cesarean delivery, the patient and her husband opted for external cephalic version.

The external cephalic version procedure was successfully performed during early labor, achieving cephalic presentation as confirmed by ultrasound, and no artificial rupture of membrane was done; the patient was kept under observation. However, within 3 h, the fetus reverted to a transverse lie, which was confirmed by another ultrasound. At that time, vaginal examination revealed advanced labor with an 8 cm cervical dilatation, bulging membranes, and no palpable presenting part, and no attempt at fetal head stabilization was done after the first external cephalic version.

An emergency cesarean section under general anaesthesia was hence performed by a specialist doctor. Intraoperatively, a poorly formed lower uterine segment was encountered, necessitating an inverted T-incision to facilitate the delivery of the baby. The baby was delivered by breech extraction with difficulty: a live female neonate weighing 3.2 kg, with Apgar scores of 9 at 1 min and 10 at 5 min, and an arterial cord pH of 7.23. Initial neonatal examination revealed a 2 cm × 4 mm scalp laceration posterior to the right ear, which was repaired with Dermabond (medical-grade topical skin adhesive), achieving satisfactory wound approximation, and the neonate was continuously observed in the neonatal intensive care unit.

**Figure 1:**
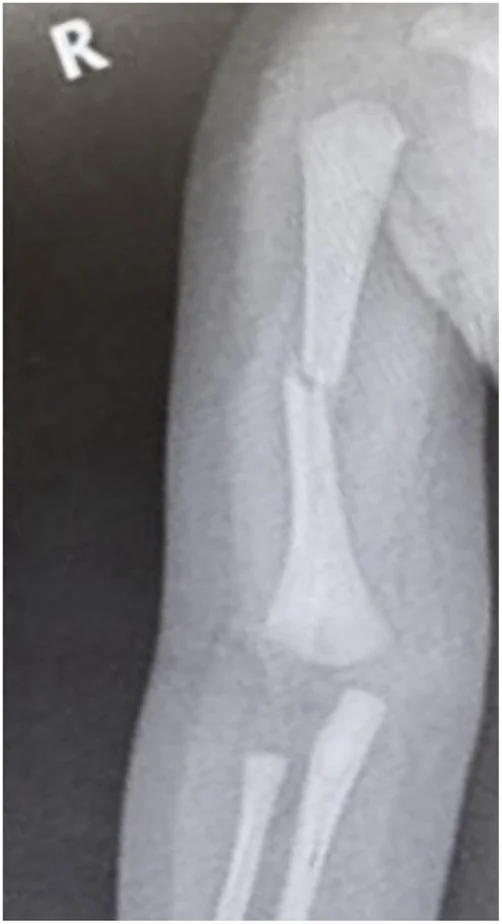
Plain radiograph demonstrating a minimally displaced midshaft fracture of the right humerus in the neonate.

Initial neonatal examination identified only the scalp laceration, with no obvious abnormalities of the extremities noted at birth. However, on postnatal day 3, right upper-limb weakness and an absent Moro reflex on the right side were observed, prompting further evaluation. X-ray subsequently confirmed a minimally displaced mid-shaft fracture of the right humerus (Figure 1). The delayed diagnosis highlights that neonatal fractures may not be clinically apparent during the immediate postnatal assessment, particularly following difficult operative deliveries. The injury was managed conservatively with immobilisation, and the infant remained clinically stable and was discharged with orthopaedic follow-up and parental education.

## Discussion

This case demonstrates the potential for neonatal injury during technically difficult cesarean delivery. Although cesarean section is generally associated with a lower risk of birth trauma than vaginal delivery, challenging fetal extraction remains a recognised cause of neonatal injury [7], 8]. In the present case, the combination of transverse lie and advanced labor created a complex operative scenario in which delivery could not be achieved through routine extraction techniques.

A major contributor to the operative difficulty was the poorly formed lower uterine segment encountered intraoperatively. In advanced labor, particularly when malpresentation is present, access to the fetus could be restricted, and extraction through a standard lower-segment uterine incision can be challenging to perform. In this case, the need for an inverted T-incision reflected the limited operative space available and the difficulty in achieving adequate access to facilitate delivery. Although extensions similar to this improve access to the fetus, they are associated with increased maternal morbidity, including greater blood loss and an increased risk of uterine rupture in subsequent pregnancies due to extension of the scar into the upper contractile portion of the uterus rather than the thinner lower segment [9], 10].

The scalp laceration and humeral fracture observed in this case were likely related to the mechanical forces required during extraction. When the fetus is not aligned with the uterine incision, as in transverse lie, delivery may need complex manoeuvres to identify and deliver the presenting fetal parts. Advanced labor further complexes these challenges by reducing uterine flexibility and limiting intrauterine space [11]. Consequently, greater manipulation and traction may be required, increasing the likelihood of both soft tissue and skeletal injury.

An important learning point from this case is that the humeral fracture was not identified during the initial neonatal examination and only became apparent on the third postnatal day following the development of right upper-limb weakness and an absent Moro reflex. This highlights the need for continued clinical surveillance post difficult cesarean section deliveries, especially when fetal manipulation or traction has been involved in the procedure. A normal examination immediately after birth does not exclude skeletal injury, and a low threshold for further investigation should be maintained when abnormal limb movement, asymmetrical reflexes, swelling, or unexplained irritability are subsequently observed in the neonate.

This case also emphasizes the importance of anticipatory surgical planning and intraoperative decision-making. Early recognition of potentially difficult extractions allows for necessary timely modification of the surgical approach, including extension of the uterine incision when required. In addition, the involvement of experienced obstetric surgeons, careful fetal handling, and avoidance of excessive traction on fetal extremities are all important strategies to minimise the risk of neonatal trauma. Awareness of these risk factors may contribute to facilitating safer delivery and improving neonatal outcomes in similarly challenging cases.

## Conclusions

This case demonstrates that neonatal trauma may occur during difficult cesarean delivery for transverse lie in advanced labor despite operative delivery. Advanced labor, malpresentation, and a poorly formed lower uterine segment contributed to a challenging extraction requiring an inverted T uterine incision. The delayed diagnosis of the humeral fracture highlights the importance of careful and repeated neonatal assessment following technically demanding deliveries. Early recognition and appropriate management of birth injuries remain essential for achieving favourable neonatal outcomes.
